# Supplementary material for: Validity of instruments to assess students' travel and pedestrian safety
Source: BMC Public Health. 2010 May 18;10:257. doi: 10.1186/1471-2458-10-257 (PMC2887818; doi:10.1186/1471-2458-10-257)
Supplement: Additional file 1 — SRTS travel survey. The English- and Spanish-language Safe Routes to School travel survey administered in written format. [file 1471-2458-10-257-S1.DOC]

**Combined Classroom Transportation Survey**

**__________________________________________**

**How did you get to school today?** Mark the one box that best shows how you got to school. Mark only one box. Carpool means that other children rode in the car besides you, including friends, neighbors, or brothers/sisters.

| Rode School  Bus | Came by ‘Carpool” | Came by Car | Rode Metro Bus | Walked with an adult | Walked without an adult | Biked |
| --- | --- | --- | --- | --- | --- | --- |
|  |  |  |  |  |  |  |

**Encuesta Combinada sobre Transporte Escolar**

**______________________________________________**

**¿Cómo llegaste a la escuela el día de hoy?** Marca el cuadro que mejor señala cómo llegaste a la escuela. Marca sólo un cuadro. Viaje compartido (o “carpool”) significa que otros niños viajaron en el mismo auto contigo, incluyendo amigos, vecinos o hermanos/hermanas.

| Tomé el autobús escolar | Llegué en auto (o carro) por “viaje compartido” | Llegué en auto (o carro) | Tomé el autobús Metro | Caminé con un adulto | Caminé sin un adulto | En bicicleta |
| --- | --- | --- | --- | --- | --- | --- |
|  |  |  |  |  |  |  |

-------------------Please do not write below this line-------------------

----------------Favor de no escribir debajo de esta línea---------------
