# Supplementary material for: Validity of instruments to assess students' travel and pedestrian safety
Source: BMC Public Health. 2010 May 18;10:257. doi: 10.1186/1471-2458-10-257 (PMC2887818; doi:10.1186/1471-2458-10-257)
Supplement: Additional file 2 — Pedestrian safety behavior checklist. Checklist of pedestrian safety behaviors assessed at major school intersections by trained observers. [file 1471-2458-10-257-S2.DOC]

**STREET CROSSING BEHAVIORS**

Use this form to assess the students’ street crossing behaviors.

| Today’s Date | | |  | | |  | |  | | | |
| --- | --- | --- | --- | --- | --- | --- | --- | --- | --- | --- | --- |
| Your Name | | |  | | |  | |  | | | |
| Location for these observations | | |  | | |  | |  | | | |
| Does this corner have a “walk / don’t walk” signal?  Does this corner have a stop sign?  Does this corner have a safety patrol or crossing guard?  Numbers of lanes of traffic at the corner: | | | | | | | | □ YES □ NO  □ YES □ NO  □ YES □ NO  □ 1; □ 2; □ 3; □ 4; □ 5; □ 6; □ 7; or □ 8 | | | |
| Nearest Elementary School | | |  | | |  | |  | | | |
| Time Started | | |  | | | Time Ended | |  | | | |
|  | | |  | | |  | |  | | | |
| **DIRECTIONS:** | | |  | | |  | |  | | | |
| Please identify the study children as follows: students in a walking school bus will be wearing bright, reflective vests and walking in a group with at least two adults. If there is no traffic signal, skip the last observation “wait or followed the traffic signal.”  An ADULT is someone grown-up or in high school.  Please DON’T COUNT students on bikes, skateboard, scooters or riding in strollers.  Please DON’T STOP students or other pedestrians to ask them questions.  **First, write in the time and indicate if the student is a part of a walking school bus (wearing a bright, reflective vest). Then, check every box that you see the student do. Leave boxes blank if the student does not do them.** | | | | | | | | | | | |
| **Time**  (write in time and circle am or pm) | Student is part of a **walking school bus** | **Crossed at corner**  or at a crosswalk | | **Crossed with adult**  or with the safety patrol | **Stopped**  at the curb | | **Looked**  left-right-left | | **Kept looking**  while crossing | **Walked**  (did not run) across the street | **Waited or followed**  the traffic signal  (if there is one) |
| am/pm | □ | □ | | □ | □ | | □ | | □ | □ | □ |
| am/pm | □ | □ | | □ | □ | | □ | | □ | □ | □ |
| am/pm | □ | □ | | □ | □ | | □ | | □ | □ | □ |
| am/pm | □ | □ | | □ | □ | | □ | | □ | □ | □ |
| am/pm | □ | □ | | □ | □ | | □ | | □ | □ | □ |
| am/pm | □ | □ | | □ | □ | | □ | | □ | □ | □ |
| am/pm | □ | □ | | □ | □ | | □ | | □ | □ | □ |
| **Time**  (write in time and circle am or pm) | Student is part of a **walking school bus** | **Crossed at corner**  or at a **crosswalk** | | **Crossed with adult**  or with the **safety patrol** | **Stopped**  at the curb | | **Looked**  left-right-left | | **Kept looking**  while crossing | **Walked**  (did not run) across the street | **Waited or followed**  the traffic signal  (if there is one) |
| am/pm | □ | □ | | □ | □ | | □ | | □ | □ | □ |
| am/pm | □ | □ | | □ | □ | | □ | | □ | □ | □ |
| am/pm | □ | □ | | □ | □ | | □ | | □ | □ | □ |
| am/pm | □ | □ | | □ | □ | | □ | | □ | □ | □ |
| am/pm | □ | □ | | □ | □ | | □ | | □ | □ | □ |
| am/pm | □ | □ | | □ | □ | | □ | | □ | □ | □ |
| am/pm | □ | □ | | □ | □ | | □ | | □ | □ | □ |
| am/pm | □ | □ | | □ | □ | | □ | | □ | □ | □ |
| am/pm | □ | □ | | □ | □ | | □ | | □ | □ | □ |
| am/pm | □ | □ | | □ | □ | | □ | | □ | □ | □ |
| am/pm | □ | □ | | □ | □ | | □ | | □ | □ | □ |
| am/pm | □ | □ | | □ | □ | | □ | | □ | □ | □ |
| am/pm | □ | □ | | □ | □ | | □ | | □ | □ | □ |
| am/pm | □ | □ | | □ | □ | | □ | | □ | □ | □ |
| am/pm | □ | □ | | □ | □ | | □ | | □ | □ | □ |
| am/pm | □ | □ | | □ | □ | | □ | | □ | □ | □ |
| am/pm | □ | □ | | □ | □ | | □ | | □ | □ | □ |
| am/pm | □ | □ | | □ | □ | | □ | | □ | □ | □ |
| am/pm | □ | □ | | □ | □ | | □ | | □ | □ | □ |
| am/pm | □ | □ | | □ | □ | | □ | | □ | □ | □ |
| am/pm | □ | □ | | □ | □ | | □ | | □ | □ | □ |
| am/pm | □ | □ | | □ | □ | | □ | | □ | □ | □ |
| am/pm | □ | □ | | □ | □ | | □ | | □ | □ | □ |
| am/pm | □ | □ | | □ | □ | | □ | | □ | □ | □ |
